# Health Anxiety and Mental Health Outcome During COVID-19 Lockdown in Italy: The Mediating and Moderating Roles of Psychological Flexibility

**DOI:** 10.3389/fpsyg.2020.02195

**Published:** 2020-08-31

**Authors:** Giulia Landi, Kenneth I. Pakenham, Giada Boccolini, Silvana Grandi, Eliana Tossani

**Affiliations:** ^1^Department of Psychology, University of Bologna, Bologna, Italy; ^2^Laboratory of Psychosomatics and Clinimetrics, Department of Psychology, University of Bologna, Cesena, Italy; ^3^School of Psychology, The University of Queensland, Brisbane, QLD, Australia

**Keywords:** health anxiety, COVID-19, pandemic, depression, anxiety, psychological flexibility, quarantine

## Abstract

The COVID-19 emergency has severely affected the Italian population. During a pandemic, those with high health anxiety are at risk of adverse mental health outcomes, including peritraumatic distress and mood disturbance. No prior research has explored the role of psychological flexibility in protecting people at high risk of poorer mental health impacts due to health anxiety during a pandemic. Psychological flexibility is the cornerstone of psychological health and resiliency. According to acceptance and commitment therapy (ACT), it involves behaving consistently with one’s chosen values even in the presence of emotional and mental discomfort. This study examined the mediating and moderating roles of psychological flexibility in the link between trait health anxiety and three mental health outcomes: COVID-19 peritraumatic distress, anxiety, and depression. We hypothesized that higher psychological flexibility would decrease the negative impacts of trait health anxiety on mental health outcomes. During the mandatory national lockdown (*M* = 35.70 days, *SD* = 8.41), 944 Italian adults (75.5% female, *M* = 38.86 years, *SD* = 13.20) completed an online survey consisting of standardized measures of psychological flexibility, trait health anxiety, COVID-19 distress, anxiety, and depression. Results indicated that psychological flexibility did not moderate the link between trait health anxiety and mental health outcomes. Rather, greater psychological flexibility mediated decreases in the adverse effects of trait health anxiety on COVID-19 distress, anxiety, and depression. In particular, two psychological flexibility processes, observing unhelpful thoughts rather than taking them literally (defusion) and values-based action (committed action), mediated decreases in the negative effects of trait health anxiety on all mental health outcomes. In contrast, the psychological flexibility process acceptance, which involves openness to inner discomfort, mediated increases in negative mental health outcomes. Overall, the combination of these processes mitigated the detrimental impacts of trait health anxiety on mental health during the emergency mandatory COVID-19 nationwide lockdown. Consistent with the ACT conceptualization of psychological flexibility, findings suggest embracing (rather than avoiding) inner discomfort and observing associated unhelpful thoughts, while also engaging in values-based action, increases resilience during adversity. Evidenced-based large-scale online public health interventions that target psychological flexibility in those experiencing health anxiety in the context of a pandemic are urgently needed. Many empirically-based ACT interventions are suited for this purpose.

## Introduction

The coronavirus disease 2019 (COVID-19) outbreak caused by SARS-CoV-2 has severely affected the Italian population which was subjected to extreme and unprecedented social distancing measures for almost 2 months (Remuzzi and Remuzzi, 2020). In order to contain the spread of COVID-19, the Italian government on March 9 implemented a national lockdown in which movements outside one’s city were forbidden and all Italians were required to stay home and refrain from any social contact with friends and relatives outside their household (Lazzerini and Putoto, 2020). Schools and universities and all “non-essential” industries and retail stores had to remain closed until May 4, and traveling was only permitted for work (where work from home was not possible), health care, or other basic necessities (e.g., obtaining groceries) (Government of Italy, 2020). Mandatory quarantine was required to reduce the exponential spread of the virus and to alleviate the pressure on the healthcare system. However, the pandemic itself and prolonged home confinement may negatively impact mental health, due to fear of contracting the disease, large-scale social isolation, and the saturation of news and social media with negative COVID-19 information (Asmundson and Taylor, 2020b; Brooks et al., 2020; Garfin et al., 2020). During the mandatory lockdown, Italy registered over 28,884 deaths due to COVID-19 (Italian Ministry of Health, 2020).

Investigation of the impacts of the pandemic on mental health has been identified as a high research priority (Holmes et al., 2020). Preliminary data suggest that elevated anxiety and depressive symptoms and sleep impairment are very common (Rajkumar, 2020; Xiao et al., 2020a, b). Two of the first studies conducted during the Chinese national lockdown indicated that 35% experienced mild to severe COVID-19 peritraumatic distress (*N* = 52,730; Qiu et al., 2020), 54% rated the psychological impact of the outbreak as moderate to severe, 16.5% endorsed moderate to severe depressive symptoms, and 28.8% moderate to severe anxiety symptoms (*N* = 1,210; Wang et al., 2020). Another study conducted on an Italian sample of 18,147 indicated that 37% of participants experienced post-traumatic stress, while 21–23% reported high anxiety, perceived stress, insomnia, and adjustment disorders (Rossi et al., 2020). In view of these data on the adverse effects of the COVID-19 pandemic on mental health, the purpose of the present study was to explore the role of psychological flexibility in protecting people at high risk of poorer mental health impacts due to elevated health anxiety during the COVID-19 pandemic and lockdown.

### Health Anxiety During a Pandemic

Given the extremely high COVID-19 infection rate and relatively high mortality, individuals with higher health anxiety are at increased risk for elevated peritraumatic stress, anxiety, and depression (Taylor, 2019; Ahorsu et al., 2020; Asmundson and Taylor, 2020a, b; Rossi et al., 2020; Wang et al., 2020). Health anxiety has been measured as state and trait, with the latter being conceptualized as a relatively stable dispositional individual difference (Taylor, 2019). It is defined as excessive awareness of one’s bodily sensations, such as those related to viral infections (e.g., fever, coughing and aching muscles), and the persistent propensity to attribute them to a sign of a severe medical condition (Salkovskis et al., 2002; Asmundson et al., 2010; Asmundson and Taylor, 2020b).

Consistent with cognitive behavior therapy theory, health anxiety symptoms occur on a continuum, from mild to severe, and contribute to hypochondriasis and other somatic and illness anxiety disorders (Fava et al., 2000; Salkovskis et al., 2002; Sirri et al., 2008; Asmundson et al., 2010; Taylor, 2019). During a pandemic people typically receive a great amount of information about the virus from the media, which is likely to intensify health anxiety in those who are vulnerable to such symptoms (Asmundson et al., 2010; Sirri et al., 2015; Garfin et al., 2020; Gao et al., 2020). In particular, people who had high trait health anxiety before the COVID-19 pandemic are likely to be at increased risk for adverse mental health outcomes, as their tendency to misinterpret bodily sensations (e.g., coughing) could evoke a profound fear of having contracted the virus (Wheaton et al., 2012; Taylor, 2019; Asmundson and Taylor, 2020a, b; Li et al., 2020; Rajkumar, 2020).

While there is some evidence of an association between higher health anxiety and greater negative mental health outcomes during a pandemic (Wheaton et al., 2012; Blakey and Abramowitz, 2017), no published study has examined the mediating or moderating roles of protective psychological factors in this relationship. Therefore, the purpose of the present study was to explore the mediating and moderating effects of psychological flexibility on the adverse impacts of health anxiety on mental health outcomes during a pandemic lockdown.

### Psychological Flexibility

Psychological flexibility is the cornerstone of psychological health and is positively related to resiliency (Kashdan and Rottenberg, 2010). A psychological flexibility model underpins one of the most promising contemporary variants of cognitive behavior therapy, Acceptance and Commitment Therapy (ACT; Hayes et al., 2012). According to the ACT model, psychological flexibility involves behaving consistently with one’s chosen values even in the presence of unwanted intrusive internal experiences such as emotional discomfort or self-critical thinking. ACT uses six interrelated core processes to increase psychological flexibility: (1) acceptance: openness to experience, (2) cognitive defusion: observing thoughts rather than taking them literally, (3) present moment awareness (mindfulness): open and responsive awareness of the present, (4) self-as-context: flexible self-awareness and perspective taking, (5) values: freely chosen personally meaningful life directions, (6) committed action: values-guided effective action.

ACT is as an empirically supported treatment for a range of mental health problems (see reviews, Hayes et al., 2006; Powers et al., 2009; Ruiz, 2010; Swain et al., 2013; A-tjak et al., 2015; Spijkerman et al., 2016). ACT has also been effective in the context of community disasters. For example, an ACT-based self-help program effectively reduced psychological distress among war refugees (Tol et al., 2020). In a randomized controlled trial, ACT intervention participants with severe health anxiety evidenced a greater reduction in symptoms compared to the control group, and these intervention effects were mediated by psychological flexibility (Eilenberg et al., 2016, 2017). In addition, lower psychological flexibility has been found to predict trauma and mental health problems in the context of natural disasters, school shootings, and violent crimes (e.g., Gold et al., 2007; Kumpula et al., 2011; Marshall and Brockman, 2016). Nevertheless, few studies have examined the mediating and moderating roles of each of the six core psychological flexibility processes on mental health outcomes, particularly during a pandemic (Rolffs et al., 2018; Makriyianis et al., 2019; Rogge et al., 2019; Stabbe et al., 2019; Lin et al., 2020).

### Nature of the Role of Psychological Flexibility in the Link Between Health Anxiety and Mental Health During a Pandemic Lockdown

As a protective factor, psychological flexibility may influence the link between health anxiety and mental health via mediating or moderating mechanisms. We found no published theoretical or empirical data on either the mediating or moderating role of psychological flexibility in the link between health anxiety and mental health outcomes in general, or in the context of a pandemic. However, in the broader literature psychological flexibility has been examined as both a mediator and a moderator (Masuda et al., 2011; Fischer et al., 2016; Novaes et al., 2018; Makriyianis et al., 2019; Ramaci et al., 2019; Pakenham et al., 2020). Studies that have examined psychological flexibility as a mediator have in the main tested models where the independent variable is typically a stable personality characteristic or a risk factor related to a personality trait (e.g., self-concealment, Masuda et al., 2011; early maladaptive schemas, Fischer et al., 2016; adverse childhood experiences, Makriyianis et al., 2019), and the dependent variable is a mental health outcome, most frequently depression or anxiety. In the only published study that has examined both the mediating and moderating roles of psychological flexibility with respect to a personality characteristic (e.g., early maladaptive schemas; Fischer et al., 2016), the mediation model was stronger than the moderation model.

Alternatively, studies that have examined psychological flexibility as a moderator have mostly tested models where the independent variable is a contextual risk factor rather than a personality characteristic, including work stressors (Ramaci et al., 2019), job demands (Novaes et al., 2018), and COVID-19 risk factors (Pakenham et al., 2020). Regarding the latter, psychological flexibility mitigated the adverse effects of COVID-19 pandemic and lockdown risk factors on mental health via a moderation pathway.

The present study examined the role of psychological flexibility, including its six processes, as mediators and moderators of the effects of health anxiety on the mental health outcomes of COVID-19 peritraumatic distress, anxiety, and depression. In this study the independent variable in the mediation model is trait health anxiety, which is closely related to personality pathology dimensions (e.g., neuroticism) (Taylor, 2019; Skjernov et al., 2020). Furthermore, given the research findings showing that trait anxiety is associated with maladaptive avoidance (Fava et al., 2000) and impaired cognitive flexibility and prefrontal control (Eysenck et al., 2007; Bishop, 2009; Park and Moghaddam, 2017; Wilson et al., 2018), we reasoned that trait health anxiety in the context of a pandemic is likely to diminish psychological flexibility, which in turn accounts for the adverse effects of health anxiety on mental health outcomes.

Given that psychological flexibility has been shown to mediate and moderate the effects of personality characteristics and contextual risk factors on mental health respectively, we predicted that psychological flexibility would emerge as a mediator rather than a moderator in the link between trait health anxiety and mental health. Specifically, we hypothesized that higher global psychological flexibility would reduce the negative impacts of trait health anxiety on mental health outcomes via a mediation rather than a moderation mechanism. We did not make specific predictions about the effects of each of the six psychological flexibility processes on the link between trait health anxiety and mental health because they are contextually sensitive, and in the context of a pandemic and lockdown it is unclear how each of these may function. However, we expected the overall impact of the six processes would result in global psychological flexibility reducing the adverse effects of trait health anxiety on mental health.

## Materials and Methods

### Participants and Recruitment Procedure

A total of 944 respondents completed an online survey during the Italian mandatory lockdown. Inclusion criteria were living in Italy and being at least 18 years of age. Exclusion criteria were living outside of Italy during lockdown and being under 18 years of age. Participants were recruited through social media (e.g., Facebook, WhatsApp, etc.) and a snowballing procedure whereby participants were asked to invite friends in similar circumstances to participate in the study. The survey was advertised as research designed to examine the psychological impacts of the COVID-19 pandemic. Recruitment information stated that participation was voluntary, anonymous, and that withdrawal from the study was possible at any time. The survey was developed with the Qualtrics software and took approximately 15–20 min to complete. Participants clicked the link in the advertisement and, after providing active online informed consent, completed the survey. Participants were required to complete an item before proceeding to the next item. Due to the online survey methodology and recruitment primary by social network, it was not possible to calculate a response rate. The study was approved by the University of Bologna ethics committee.

### Measures

#### Demographics

Participants indicated their age (date of birth), gender (female vs. male), education (elementary school, middle school, high school diploma, bachelor’s degree, master’s degree, specialization, or PhD), marital status (single, married/in domestic partnership, widowed, separated/divorced), employment (employed, unemployed, student, retired) and ethnicity (Italian: yes/no or specify). To gauge socio-economic status, participants were asked to indicate whether they were below, average, or above the mean income of the population.

#### COVID-19 Lockdown Variables

The following information was obtained on participants’ lockdown experiences: number of days in lockdown, number of people in the household, living alone during lockdown, perception of available personal space (i.e., “Is the size of your home enough to guarantee your personal space, despite the mandatory lockdown, such as number of rooms in relation to the people you live with?” rated on a 5-point Likert scale from 1 = *not at all* to 5 = *very much*), lost work or in redundancy fund because of lockdown, COVID-19 infection in self and other people (family members, close others, roommates, or friends), severity of COVID-19 symptoms (rated on a 5-point Likert scale from 1 = *not at all serious* to 5 = *very serious*), hospitalization of significant others (family members, close others, roommates, or friends), and death of loved ones due to COVID-19.

#### Trait Health Anxiety

The trait version of the Short Health Anxiety Inventory (SHAI; Salkovskis et al., 2002), a self-report questionnaire composed of 18 items, was used to assess trait health anxiety. Each item presents a specific health anxiety symptom, such as worry about health, awareness of bodily sensations or changes, and feared consequences of having an illness. Participants rated the frequency of their anxiety symptom during the last 6 months on a 4-point scale (0 = “I do not worry about my health,” 1 = “I occasionally worry about my health,” 2 = “I spend much of my time worrying about my health,” and 3 = “I spend most of my time worrying about my health”). Items are summed, with higher scores indicating higher trait health anxiety (range 0–54). A cut-off score of 18 has been commonly used to indicate a moderate level of trait health anxiety, while a score of 27 indicates a higher probability of meeting *DSM-IV* criteria for hypochondriasis (Alberts et al., 2013). For the purpose of this study, the SHAI scale was translated into Italian by a bilingual translator and two authors of this report. The SHAI has shown sound psychometric properties including good reliability and validity in clinical and non-clinical populations (Salkovskis et al., 2002; Abramowitz et al., 2007; Alberts et al., 2013). Because the SHAI has not been validated in Italian, we ran a confirmatory factor analysis (CFA) with the robust maximum likelihood estimator (MLR; Muthén and Muthén, 1998–2018). Fit indices of the CFA of the Italian SHAI were satisfactory for the original one-factor model: χ^2^ (129) = 409.117, *p* < 0.001; CFI = 0.925; TLI = 0.911; RMSEA = 0.048; RMSEA CI = [0.043, 0.053]; SRMR = 0.043 (factor loadings are reported in Supplementary Table A). The Italian SHAI demonstrated good internal consistency (α = 0.84) in the current sample.

#### Psychological Flexibility

We used the psychological flexibility dimension of The Multidimensional Psychological Flexibility Inventory (MPFI; Rolffs et al., 2018) to assess psychological flexibility and its constituent six core processes: Acceptance (e.g., “I tried to make peace with my negative thoughts and feelings rather than resisting them” and “I opened myself to all of my feelings, the good and the bad”), Present Moment Awareness (e.g., “I was in tune with my thoughts and feelings from moment to moment” and “I strived to remain mindful and aware of my own thoughts and emotions”), Self-as-context (e.g., “Even when I felt hurt or upset, I tried to maintain a broader perspective” and “When something painful happened, I tried to take a balanced view of the situation”), Defusion (e.g., “I was able to step back and notice negative thoughts and feelings without reacting to them” and “When I was scared or afraid, I was able to gently experience those feelings, allowing them to pass”), Values (e.g., “I was very in-touch with what is important to me and my life” and “My deeper values consistently gave direction to my life”), and Committed Action (e.g., “Even when I stumbled in my efforts, I didn’t quit working toward what is important” and “I didn’t let my own fears and doubts get in the way of taking action toward my goals”). Participants rated the extent to which they agreed with each item on a 6-point Likert scale ranging from 1 (*never true*) to 6 (*always true*). Scores are averaged and higher scores indicate higher flexibility on the global psychological flexibility score and on the six psychological flexibility processes. The Italian version of this scale is currently under validation by some authors of this report. The MPFI has demonstrated good reliability and validity in clinical and non-clinical samples (Lin et al., 2020; Rogge et al., 2019; Stabbe et al., 2019). In the derivation study, the Cronbach’s alpha for the global psychological flexibility scale was 0.91 and the alpha in the present study was 0.94. Individual subscales also had high alphas in the original investigation (ranging from 0.89 to 0.93) and the range in the present study was 0.85–0.94. Because the MPFI has not yet been validated in Italian, we ran a CFA with the robust maximum likelihood estimator (MLR; Muthén and Muthén, 1998–2018) on the psychological flexibility dimension of the instrument. Fit indices for the original six-factor model were satisfactory: χ^2^(397) = 1542.769, *p* < 0.001; CFI = 0.937; TLI = 0.931; RMSEA = 0.055; RMSEA CI = [0.052, 0.058]; SRMR = 0.084 (factor loadings are reported in Supplementary Table B).

#### COVID-19 Peritraumatic Distress

The COVID-19 Peritraumatic Distress Index (CPDI; Qiu et al., 2020) is a self-report questionnaire composed of 24 items that assess COVID-19 peritraumatic distress symptoms. The measure was developed to evaluate COVID-19 distress in China. The English version was made available by the authors of the measure and was translated into Italian by a bilingual translator and two authors of this report. Items examine the frequency of anxiety, depression, specific phobias, cognitive change, avoidance, compulsive behavior, physical symptoms, and loss of social functioning in the past week (e.g., “I can’t stop myself from imagining myself or my family being infected and feel terrified and anxious about it,” “I feel empty and helpless no matter what I do,” and “During this COVID-19 period, I often feel dizzy or have back pain and chest distress”). Items are rated on a 5-point Likert scale ranging from 0 (*never*) to 4 (*most of the time*). Scores are summed (total score range 0–100) with higher scores indicating higher COVID-19 peritraumatic distress. Chinese normative data revealed the following ranges for the total score: 28–51 mild to moderate distress and ≥ 52 severe distress. The CPDI demonstrated satisfactory reliability and content validity in the derivation study (Qiu et al., 2020). Because the CPDI has not been validated in Italian, we ran a CFA with the robust maximum likelihood estimator (MLR; Muthén and Muthén, 1998–2018). Fit indices of the CFA of the Italian CPDI were adequate for a one-factor model: χ^2^(276) = 6,307.124, *p* < 0.001; CFI = 0.914; TLI = 0.900; RMSEA = 0.048; RMSEA CI = [0.044, 0.052]; SRMR = 0.047. However, five items did not comply with the item loading criteria ≥ 0.32 and were eliminated (items 5, 8, 9, 10, and 11). A second CFA was conducted on the remaining 19 items leading to a one-factor solution with satisfactory fit: χ^2^(146) = 487.400, *p* < 0.001; CFI = 0.933; TLI = 0.922; RMSEA = 0.050; RMSEA CI = [0.045, 0.055]; SRMR = 0.040 (factor loadings are reported in Supplementary Table C). The Cronbach’s alpha for the final Italian CPDI was 0.90 in this sample.

#### Anxiety

The General Anxiety Disorder Scale (GAD-7; Spitzer et al., 2006) is a widely used self-report questionnaire measuring general anxiety symptoms over the past 2 weeks. It is composed of 7 items (e.g., “Not being able to stop or control worrying”) evaluated on a 4-point Likert scale, ranging from 0 (*not at all*) to 3 (*nearly every day*). Scores are summed with higher scores indicating higher anxiety symptoms. We used the Italian version of the GAD-7 developed by the MAPI Research Institute (Kroenke and Spitzer, 2010). This measure has good psychometric properties (Löwe et al., 2008; Plummer et al., 2016). The Cronbach’s alpha in this sample was 0.90. Normative data show the following ranges for the total score: minimal (0–4), mild (5–9), moderate (10–14), and severe (15–21) anxiety symptoms (Spitzer et al., 2006).

#### Depression

The Patient Health Questionnaire (PHQ-9; Spitzer et al., 1999) is a widely used self-report measure of depressive symptomatology over the past 2 weeks. It is composed of 9 items (e.g., “Feeling down, depressed, or hopeless”) evaluated on a 4-point Likert scale, from 0 (*not at all*) to 3 (*nearly every day*). Scores are summed with higher scores indicating higher depression, ranging from minimal (0–4), mild (5–9), moderate (10–14), moderately severe (15–19), to severe (20–27) levels of depressive symptoms. We used the Italian validated version of the PHQ-9 (Mazzotti et al., 2003). This measure has demonstrated sound psychometric properties (Manea et al., 2012). Cronbach’s alpha in this sample was 0.87.

### Data Analysis

All analyses were performed in IBM SPSS 24 using the Process macro v.3.4. Regression diagnostics were conducted according to the recommendations of Darlington and Hayes (2017). Outliers were identified using *t*-residual distributions. Pearson’s and Spearman’s correlations were conducted between trait health anxiety, psychological flexibility and its six processes, all mental health outcomes, and potential confounding variables (i.e., gender and age), which we later controlled for in mediation and moderation analyses. We also reported descriptive data on levels of trait health anxiety, anxiety, and depression relative to norms. For the Italian modified CPDI, we calculated the mean and *SD* for the total sample. Participants who scored one *SD* above the mean were deemed to fall in the mild to moderate range of clinically significant COVID-19 distress and those who scored two *SDs* above the mean were regarded as falling in the severe range of clinically significant COVID-19 distress. To test the mediational role of psychological flexibility (M) in the link between trait health anxiety and all mental health outcomes, three simple mediational analyses were performed with Process Model 4 (Hayes, 2018), one for each dependent variable (i.e., COVID-19 peritraumatic distress, anxiety, and depression). Process Model 4 enables testing of the direct and indirect effects with a single mediator or multiple mediators in parallel (Hayes, 2018). Indirect effects were analyzed by computing bias-corrected 99% confidence intervals (CIs) with 10,000 random bootstrap samples: statistical significance of the indirect effects was established when zero was not included in the lower and upper levels of the CIs (Hayes, 2018). To test the moderating role of psychological flexibility (W) in the link between trait health anxiety and mental health outcomes, three simple moderation analyses were performed with Process Model 1 (Hayes, 2018), one for each dependent variable. Process Model 1 enables testing the conditional effect (i.e., the effect of one variable on another, conditioned on a third or interaction) by estimating the effect of X on Y at a certain point (or points) along the moderator, and testing whether this effect is significant. Statistical significance of simple moderations was established when the 99% CIs for the interaction (trait health anxiety × moderator) did not include zero (Hayes, 2018). Finally, models in which psychological flexibility emerged as a significant mediator or moderator were further examined using the six psychological flexibility processes, instead of the global psychological flexibility score. Given the primary hypothesis regarding the mediating and moderating effects of global psychological flexibility was tested across three mental health outcomes, more stringent significance levels of *p* < 0.01 and 99% CIs were used for these analyses to control for Type I error. The subsequent mediation or moderation analyses conducted on the six psychological flexibility processes were more exploratory in nature and thus, the conventional significance levels of *p* < 0.05 and 95% CIs were retained.

## Results

### Sample Characteristics

Three cases were identified as outliers. Exclusion of the outliers did not change the results of the primary analyses, hence, analyses are reported using the full sample. The sample was composed of 944 Italian adults, 75.3% female, aged 18–81 (*M* = 38.8, *SD* = 13.2). Almost all participants (98.4%) were of Italian nationality. Thirteen participants were of German (*n* = 2), Romanian (*n* = 2), Swiss (*n* = 2), Albanian (*n* = 1), Argentina (*n* = 1), Ecuadorian (*n* = 1), Lebanese (*n* = 1), Palestinian (*n* = 1), Slovenian (*n* = 1), and Ukrainian (*n* = 1) nationality. Regarding highest level of education, approximately half of the sample (48.1%) had a bachelor’s degree, 26.4% completed high school, and 22.1% postgraduate courses. Almost half (46%) of the sample were either married or living with a partner, while 54% were single, widowed, or divorced. Regarding socioeconomic status, 81.3% endorsed the middle socioeconomic band, 10.6% average, and 8.2% wealthier than the average. Most (66.4%) participants were employed, 11.2% were students, and 9.6% unemployed.

Table 1 summarizes the COVID-19 context of the sample. Participants spent on average 35.70 days in lockdown (*SD* = 8.41) and lived with a mean of 2.55 cohabitants (*SD* = 1.21), while 23.3% lived alone during lockdown. A total of 24.3% of participants lost work or were put on a redundancy fund because of the mandatory lockdown. A total of 178 participants (18.9%) reported having been infected by COVID-19 with an average symptom severity of 1.80 (*SD* = 0.90, range 1–5). A quarter of the sample (25.5%) reported having significant others (family members, close others, roommates, or friends) infected by COVID-19, 20.6% of them were hospitalized, and 16.7% died due to COVID-19.

**TABLE 1:** Descriptive data on demographics and COVID-19 lockdown variables.

Variable	% (*n*)	*M* (*SD*)	Range
**Demographics**			
Age years		38.86 (13.20)	18.87–81.03
Gender: female	73.5 (694)		
Currently working	66.4 (627)		
Currently studying	11.2 (106)		
Currently unemployed	9.6 (90)		
Retired	5.3 (50)		
**COVID-19 lockdown variables**			
Days in lockdown		35.70 (8.41)	10–90
Number of cohabitants		2.55 (1.21)	1–6
Living alone	23.3 (220)		
Perception of personal space^*a*^		3.70 (1.01)	1–5
Loss of work or receiving redundancy fund	24.3 (229)		
COVID-19 infected	18.9 (178)		
Severity of COVID-19 symptoms^*b*^		1.80 (0.90)	1–5
Family member infected	7.5 (71)		
Family member hospitalized	2.6 (25)		
Family member deceased	2.2 (21)		

**^*a*^Rated on a 5-point Likert scale from 1 (not at all) to 5 (very much). ^*b*^Rated on a 5-point Likert scale from 1 (not at all serious) to 5 (very serious).**

Regarding descriptive data on trait health anxiety, 33.8% of the sample reported moderate symptomatology, while 8.1% reached severe levels associated with a higher probability of meeting *DSM-IV* criteria for hypochondriasis. Considering the mental health outcomes, 10.3% of participants reported mild to moderate COVID-19 peritraumatic distress (1 *SD* above the mean), while 5.2% had severe levels of symptomatology (2 *SDs* above the mean). With respect to anxiety, 11.5 and 6.6% of the sample reported moderate and severe levels of symptomatology, respectively. A total of 14.6% of participants experienced moderate levels of depressive symptomatology, while 8.8% fell in the severe depression range.

### Correlations Among Trait Health Anxiety, Psychological Flexibility, Mental Health Outcomes, and Demographics

Pearson’s and Spearman’s correlations were conducted for continuous or categorical variables, respectively, in order to investigate the relationships between trait health anxiety, psychological flexibility, mental health outcomes, and demographics (see Table 2). The correlations between higher trait health anxiety and poorer outcomes on all mental health variables were significant and of a moderate magnitude. Lower trait health anxiety was significantly, albeit weakly, correlated with higher global psychological flexibility. Four of the psychological flexibility processes were significantly related to lower trait health anxiety. Present moment awareness was unrelated to trait health anxiety and acceptance was weakly but significantly associated with higher trait health anxiety. Global psychological flexibility and all psychological flexibility processes were related to better outcomes on all mental health variables except acceptance, which was significantly but weakly correlated with higher COVID-19 peritraumatic distress, anxiety, and depression. The six psychological flexibility processes were significantly positively correlated with higher global psychological flexibility. All mental health outcomes were positively and strongly correlated with each other. Of the demographics, only gender and age were significantly but weakly associated with trait health anxiety and all mental health outcomes. Specifically, being female and younger was significantly related to higher trait health anxiety and poorer mental health outcomes.

**TABLE 2:** Descriptive data and correlations among trait health anxiety, psychological flexibility, mental health outcomes, gender, and age (*N* = 944).

%	*M* (*SD*)	Range	α	1	2	2a	2b	2c	2d	2e	2f	3	4	5	6
1. Trait health anxiety	16.81 (6.64)	2–45	0.84	–											
2. Global psychological flexibility	3.66 (0.90)	1–6	0.85	−0.21**	–										
2a. Acceptance	2.85 (1.08)	1–6	0.85	0.10**	0.53**	–									
2b. Present moment awareness	3.66 (1.20)	1–6	0.93	−0.04	0.76**	0.48**	–								
2c. Self as context	3.69 (1.20)	1–6	0.93	−0.22**	0.85**	0.34**	0.59**	–							
2d. Defusion	3.40 (1.19)	1–6	0.91	−0.31**	0.81**	0.26**	0.48**	0.75**	–						
2e. Values	4.28 (1.19)	1–6	0.92	−0.20**	0.81**	0.24**	0.50**	0.58**	0.58**	–					
2f. Committed action	4.12 (1.25)	1–6	0.94	−0.24**	0.78**	0.17**	0.41**	0.59**	0.60**	0.73**	–				
3. COVID-19 distress	17.95 (11.50)	0–63	0.90	0.50**	−0.34**	0.09**	−0.12**	−0.32**	−0.45**	−0.32**	−0.39**	–			
4. Anxiety	5.83 (4.55)	0–21	0.90	0.53**	−0.32**	0.10**	−0.09**	−0.32**	−0.47**	−0.30**	−0.35**	0.81**	–		
5. Depression	6.85 (5.00)	0–26	0.87	0.44**	−0.33**	0.11**	−0.09**	−0.33**	−0.43**	−0.34**	−0.41**	0.80**	0.79**	–	
6. Gender^*a*^ 75.28				−0.12**	−0.02	−0.16**	−0.05	−0.00	0.06	−0.04	0.02	−0.19**	−0.18**	−0.19**	–
7. Age	38.85 (13.43)	38.86 (13.20)		−0.13**	0.09**	−0.14**	0.05	0.09**	0.15**	0.15**	0.11**	−0.07*	−0.18**	−20**	0.07*

**^∗^p < 0.05, ^∗∗^p < 0.01. Gender: 0 = female, 1 = male. ^*a*^Spearman’s Correlation.**

### Mediating Role of Global Psychological Flexibility in the Link Between Trait Health Anxiety and Mental Health Outcomes During a Pandemic Lockdown

**FIGURE 1:**
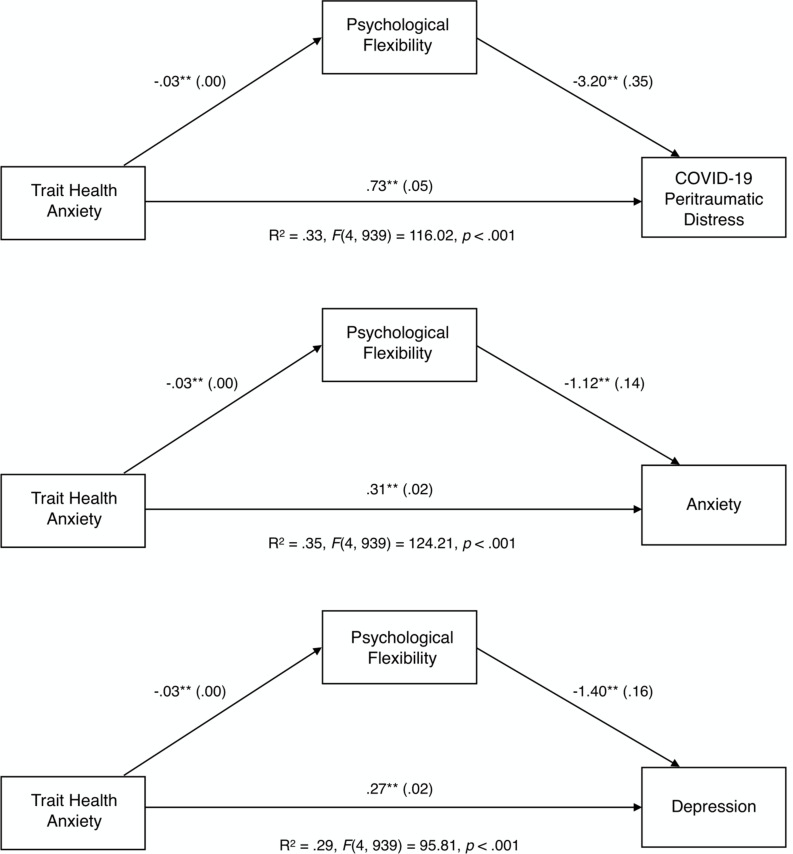
Unstandardized path coefficients (and standard errors) depicting the mediational role of global psychological flexibility between trait health anxiety and mental health outcomes. **p < 0.01.

Results of mediation analyses indicated that global psychological flexibility significantly mediated the relationship between trait health anxiety and all three mental health outcomes (indirect effect for COVID-19 peritraumatic distress: ab = 0.090, *SE* = 0.018, 99% CI [0.048, 0.142]; indirect effect for anxiety: ab = 0.031, *SE* = 0.018, 99% bootstrap CI [0.016, 0.050], and indirect effect for depression: ab = 0.031, *SE* = 0.007, 99% bootstrap CI [0.016, 0.050]). Participants with higher trait health anxiety reported lower global psychological flexibility (a = −0.028, *SE* = 0.004), which in turn decreased mental health outcomes (COVID-19 peritraumatic distress: b = −0.3.199, *SE* = 0.350; anxiety: b = −1.120, *SE* = 0.137; depression: b = −1.398, *SE* = 0.157). Trait health anxiety also directly influenced the three mental health outcomes independent of this mechanism (total effect for COVID-19 peritraumatic distress: c’ = 0.823, *SE* = 0.049, 99% CI = [0.697, 0.949]; total effect for anxiety: c’ = 0.342, *SE* = 0.019, 99% CI = [0.293,0.391]; total effect for depression: c’ = 0.31o, *SE* = 0.022, 99% CI = [0.254, 0.366]). Each model explained between 34.6% (anxiety) and 29.0% (depression) of the variance. The three simple mediational models showing that global psychological flexibility mediates the relationship between trait health anxiety and COVID-19 peritraumatic distress, anxiety, and depression are summarized in Figure 1. In each model, higher psychological flexibility reduced the detrimental impacts of trait health anxiety on all mental health outcomes.

#### Mediating Role of Psychological Flexibility Processes in the Link Between Trait Health Anxiety and Mental Health Outcomes

**FIGURE 2:**
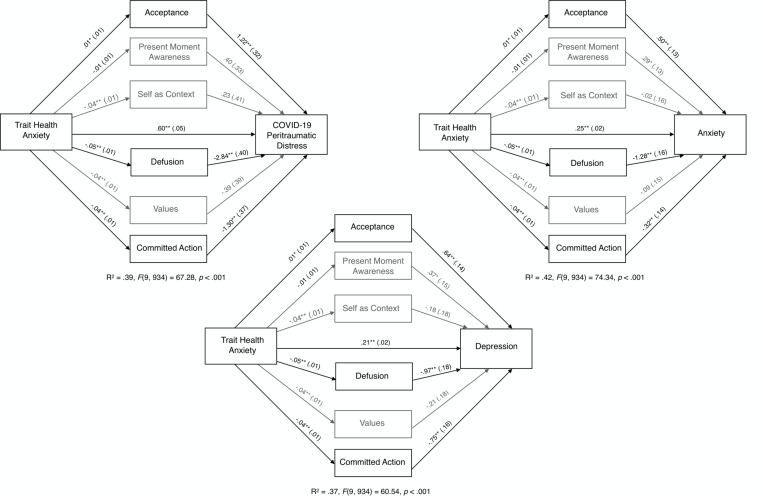
Unstandardized path coefficients (and standard errors) depicting the mediational role of the six psychological flexibility processes between trait health anxiety and mental health outcomes. Gray indicates, non-significant mediation paths; *p < 0.05. **p < 0.01.

Because global psychological flexibility emerged as a significant mediator in the relationship between trait health anxiety and each of the three mental health outcomes, we further explored the mediating role of each of the six psychological flexibility processes using parallel mediator models (M_1_ = acceptance; M_2_ = present moment awareness: M_3_ = self-as-context, M_4_ = defusion, M_5_ = values, M_6_ = committed action). Results showed that three of the six psychological flexibility processes (acceptance, defusion, and committed action) significantly mediated the relationship between trait health anxiety and all three mental health outcomes. Specifically, defusion and committed action mediated decreases in the adverse effects of trait health anxiety on the mental health outcomes, whereas acceptance mediated increases in the negative effects of health anxiety on mental health. Each model explained between 41.7% (anxiety) and 36.8% (depression) of the variance. These parallel mediational models examining the six psychological flexibility processes as mediators of the relationship between trait health anxiety and COVID-19 peritraumatic distress, anxiety, and depression are displayed in Table 3 and Figure 2.

**TABLE 3:** Indirect effects of psychological flexibility processes in the relationship between trait health anxiety and mental health outcomes.

	COVID-19 distress			Anxiety			Depression		
			
	Coeff	*SE*	95% CI	Coeff	*SE*	95% CI	Coeff	*SE*	95% CI
Total indirect effect	**0.225**	0.028	0.172, 0.282	**0.090**	0.011	0.070, 0.112	**0.104**	0.013	0.079, 0.132
Acceptance	**0.013**	0.007	0.000, 0.027	**0.005**	0.003	0.000, 0.012	**0.007**	0.004	0.000, 0.014
Present moment awareness	−0.003	0.004	−0.014, 0.003	−0.002	0.002	−0.007, 0.002	−0.002	0.003	−0.009, 0.002
Self as context	−0.009	0.017	−0.043, 0.025	0.001	0.007	−0.013, 0.015	0.007	0.008	−0.008, 0.023
Defusion	**0.153**	0.027	0.104, 0.209	**0.069**	0.011	0.048, 0.092	**0.052**	0.011	0.033, 0.075
Values	0.014	0.015	−0.015, 0.046	0.003	0.006	−0.008, 0.015	0.007	0.007	−0.006, 0.022
Committed action	**0.057**	0.018	0.024, 0.096	**0.014**	0.007	0.001, 0.028	**0.033**	0.009	0.017, 0.053

**Coeff, unstandardized coefficient of the indirect effect; SE, standard error; CI, 95% confidence interval based on 10,000 bootstrap samples. Significant mediations are displayed in bold.**

### Moderating Role of Psychological Flexibility in the Link Between Trait Health Anxiety and Mental Health Outcomes During a Pandemic Lockdown

To investigate global psychological flexibility as a moderator of the effects of trait health anxiety on the mental health outcomes, three simple moderation analyses were conducted. Results indicated that the interaction between trait health anxiety and global psychological flexibility was not significant for each of the three mental health outcomes (COVID-19 peritraumatic distress: interaction coefficient for trait health anxiety and psychological flexibility, b_3_ = 0.018, *SE* = 0.048, *F*(1, 938) = 0.132, *p* = 0.717, 99% CI [−0.107, 0.142], Δ*R*^2^ = 0.000; anxiety: interaction coefficient for trait health anxiety and psychological flexibility, b_3_ = 0.015, *SE* = 0.019, *F*(1, 938) = 0.654, *p* = 0.419, 99% CI [−0.033, 0.064], Δ*R*^2^ = 0.000; depression: interaction coefficient for trait health anxiety and psychological flexibility, b_3_ = −0.011, *SE* = 0.022, *F*(1, 938) = 0.280, *p* = 0.597, 99% CI [−0.067, 0.044], Δ*R*^2^ = 0.000). In summary, results indicate that the impact of trait health anxiety on mental health outcomes during a pandemic lockdown is not conditional on the levels of psychological flexibility.

## Discussion

Results from the present study supported our prediction that psychological flexibility would mediate decreases in the adverse effects of trait health anxiety on mental health during the COVID-19 lockdown in Italy. As expected, global psychological flexibility did not moderate the link between trait health anxiety and mental health outcomes. Examination of the six psychological flexibility processes showed that three mediated the relationship between trait health anxiety and the mental health outcomes. Specifically, defusion and committed action mitigated the adverse effects of trait health anxiety on all mental health outcomes, whereas acceptance mediated an increase in the negative effects of trait health anxiety on mental health.

The associations between higher psychological flexibility and better mental health outcomes in the present study are consistent with findings in the broader literature on psychological flexibility (Hayes et al., 2006; Kashdan and Rottenberg, 2010) and are aligned with results showing lower psychological flexibility predicts trauma and mental health problems in the aftermath of community crises such as school shootings and devastating storms (e.g., Kumpula et al., 2011; Marshall and Brockman, 2016). Findings from the present study that highlight psychological flexibility decreases the adverse effects of trait health anxiety on mental health are in accord with results from a randomized controlled trial that showed psychological flexibility mediated the beneficial effects of an ACT intervention on participants’ health anxiety symptoms (Eilenberg et al., 2016, 2017). Given that a pandemic and the associated lockdown are likely to exacerbate distress in people vulnerable to elevated health anxiety, it is noteworthy that psychological flexibility demonstrated a protective role in such an anxiety provoking context.

Results from this study showed that defusion and committed action mediated decreases in the negative effects of trait health anxiety on all mental health outcomes. Defusion involves observing unwanted thoughts and feelings and allowing them to pass, which mitigates the distress that is evoked by clinging to or struggling with inner discomfort (Hayes et al., 2012). For example, if a person has the thought “I must have the virus because I coughed” and takes it as literally true and gets absorbed in such thinking, he or she is likely to become anxious about being infected, whereas if the person views the thought for what it is, just thinking, and allows it to pass, their anxiety is less likely to intensify. In addition, they are more likely to respond adaptively in the long-term because they are able to identify mental health anxiety triggers and refrain from reactively engaging in rumination or avoidance (Eilenberg et al., 2016, 2017; Spinhoven et al., 2016). In turn, because defusion frees up cognitive-affective resources, people are more able to reflect and find meaning in the adversity of a national pandemic lockdown. Defusion also frees up energy to invest in values-based action (i.e., committed action), the second protective psychological flexibility process identified in the present study.

Committed purposeful values-based action moves a person toward a deeper connection with their personal values, even in the face of a setback such as a pandemic lockdown (Hayes et al., 2012). The pursuit of values informed goals brings fulfillment, whereas inaction, impulsivity, non-functional actions, or persistent avoidant behaving intensifies distress and leads to discontent (Hayes et al., 2012).

In contrast to the beneficial mediating effects of defusion and committed action, acceptance increased the adverse effects of trait health anxiety on mental health outcomes. Acceptance involves being open to inner experiencing (e.g., unpleasant thoughts, feelings, urges, and bodily sensations) and giving it space to organically unfold and pass. Therefore, engaging in acceptance sensitizes a person to their inner discomfort and this may account for why acceptance was related to increases in the adverse effects of trait health anxiety on mental health. According to the ACT psychological flexibility model and empirical data, in the long-term acceptance is more beneficial than experiential avoidance, which is consistently related to psychopathology (Chawla and Ostafin, 2007). The inherent adversities in a pandemic and lockdown are likely to evoke understandable and reasonable concerns about health, mortality, safety, finances, attachments, and isolation. However, consistent with ACT interventions and the ACT conceptualization of psychological flexibility, the goal is not to decrease distress, but to notice and acknowledge its presence with openness, while at the same time pursuing personal values, which in turn promotes mental health (Hayes, 2019). Hence, in the present study as predicted the overarching construct psychological flexibility was associated with better mental health outcomes, and it mediated decreases in the adverse effects of trait health anxiety on mental health. These findings are consistent with many studies that show psychological flexibility is associated with resilience and post-traumatic growth during adversity (Eakman et al., 2016; Hawkes et al., 2014).

Although values, self-as-context, and present moment awareness were significantly associated with better mental health at the bivariate level, these psychological flexibility processes did not emerge as significant mediators or moderators in the link between trait health anxiety and the mental health outcomes. However, in another study values and self-as-context significantly moderated the adverse effects of COVID-19 risk factors on mental health, and the inverse of present moment awareness exacerbated the negative impacts of these contextual factors (Pakenham et al., 2020). It is likely that the prominence and roles of the six contextually sensitive and dynamic psychological flexibility processes will vary according to the nature of the corresponding independent variables and situational factors investigated within a given model. It is only in recent years that researchers have begun to examine the roles of the individual psychological flexibility and inflexibility sub-processes in shaping mental health. Further research into how their roles vary across real-life contexts, samples, and models is required.

Our descriptive data on the levels of mental health problems in the present sample are in line with data from other studies that have examined the mental health impacts of COVID-19 lockdowns using the same measures employed in the present study. Overall, this body of data suggests that 17–54% of the general population have experienced moderate to severe levels traumatic distress, 18–29% anxiety symptoms, and 17–23% depressive symptoms (Ireland: Hyland et al., 2020; China: Qiu et al., 2020; Wang et al., 2020; Italy: Rossi et al., 2020).

In view of the adverse mental health impacts of COVID-19 and associated lockdowns and of the lingering negative psychosocial effects of prior pandemics (e.g., SARS; Hawryluck et al., 2004; Taylor, 2019), it is essential that effective public health interventions are developed to bolster resilience and promote wellbeing during and in the aftermath of such health crises. Such interventions should target psychological flexibility given the findings from the present study and those from other research indicating that psychological flexibility moderates the adverse impacts of COVID-19 contextual risk factors (Pakenham et al., 2020). Public health ACT-based interventions designed to strengthen psychological flexibility have been shown to promote mental health in a variety of populations using flexible modes of delivery in various contexts: university students via online delivery (Viskovich and Pakenham, 2020), cancer patients via phone (Hawkes et al., 2014), Sudanese refugees using audio-recorded stress-management workshops and a self-help book (Tol et al., 2020), and health anxiety patients via group delivery (Eilenberg et al., 2016). An advantage of psychological flexibility informed interventions is that they have been shown to cultivate skills that foster resilience in the context of health-related adversities, such as chronic disease (e.g., multiple sclerosis, Giovannetti et al., 2020; diabetes, Ryan et al., 2020), and to mediate the beneficial effects of these programs (Pakenham et al., 2018).

### Limitations and Future Research

Findings need to be tempered by considering the following study limitations. First, all data were collected via an online survey and self-report measures. Additional assessment methods such as structured interviews might provide more comprehensive information about the mental health impacts of the pandemic. Second, the study used a cross-sectional design and, hence, the causal directions among trait health anxiety, psychological flexibility, and mental health outcomes remain ambiguous. Longitudinal research is required to examine causal links among these variables over time. Third, convenience sampling and the bias toward female participants limits the generalizability of findings. Fourth, the three mental health outcome measures were highly inter-correlated (range 0.76–0.80), which may account for the similarity in findings across outcomes. Finally, we did not examine the potential personal growth that may be triggered by health-related adversities (Pakenham, 2011) or the wellbeing dimension of mental health. Future research should examine factors that foster benefit finding and wellbeing in the context of the COVID-19 pandemic. Notwithstanding these limitations, this study is the first to evaluate the protective role of psychological flexibility in the link between trait health anxiety and COVID-19 peritraumatic distress, anxiety, and depression.

## Conclusion

Results from the present study showed that two psychological flexibility processes, defusion and committed action, mediated decreases in the negative effects of trait health anxiety on mental health, while acceptance mediated increases in the adverse effects of trait health anxiety. Overall the combination of these processes mitigated the detrimental impacts of trait health anxiety on mental health during the emergency mandatory COVID-19 nationwide lockdown in Italy. Consistent with the ACT conceptualization of psychological flexibility, findings suggest embracing (rather than avoiding) inner discomfort and observing associated unhelpful thoughts while also engaging in values-based action increases resilience during adversity. These results indicate that public health interventions targeting psychological flexibility are likely to mitigate some of the adverse effects that high trait health anxiety has on mental health during a pandemic. Furthermore, targeting psychological flexibility in public health interventions has been identified as a viable means of improving a wide range of health outcomes in the general community (Gloster et al., 2017). Given that research into the longer-term mental health impacts of prior pandemics show lingering elevated trauma, anxiety, and depressive symptoms (e.g., after the SARS quarantine; Hawryluck et al., 2004; Taylor, 2019), it is anticipated that when this pandemic abates, mental health services will face significant demands. The evidence emerging from the burgeoning literature on psychological flexibility (see reviews Coto-Lesmes et al., 2019; Apolinário-Hagen et al., 2020; Bai et al., 2020) provides strong support for the use of ACT-based interventions to promote psychological flexibility and mental health during the COVID-19 pandemic (Gloster et al., 2017; Polizzi et al., 2020; Presti et al., 2020).

## Data Availability Statement

The raw data supporting the conclusions of this article will be made available by the authors, without undue reservation, to any qualified researcher.

## Ethics Statement

The studies involving human participants were reviewed and approved by the University of Bologna ethics committee. The patients/participants provided their written informed consent to participate in this study.

## Author Contributions

GL and GB conducted the online survey and analyzed the data. KP and ET provided critical editing and feedback on draft manuscripts. All authors contributed to the conceptualization, data interpretation, and drafting of this manuscript.

## Conflict of Interest

The authors declare that the research was conducted in the absence of any commercial or financial relationships that could be construed as a potential conflict of interest.
