# Supplementary material for: Health Anxiety and Mental Health Outcome During COVID-19 Lockdown in Italy: The Mediating and Moderating Roles of Psychological Flexibility
Source: Front Psychol. 2020 Aug 31;11:2195. doi: 10.3389/fpsyg.2020.02195 (PMC7488226; doi:10.3389/fpsyg.2020.02195)
Supplement: Supplementary file 1 [file Data_Sheet_1.PDF]

Table A. Confirmatory Factor Analysis of the Italian Short Health Anxiety Inventory (SHAI).

Items	Factor Loadings
1. I spend much of my time worrying about my health	.559
2. I notice aches/pains more than most other people (of my age)	.422
3. I am often aware of bodily sensations or changes	.353
4. I try to resist thoughts of illness but am often unable to do so	.434
5. I am often afraid that I have a serious illness	.708
6. I frequently have images of myself being ill	.603
7. I often have difficulty in taking my mind off thoughts about my health	.669
8. I am initially relieved but the worries always return later	.584
9. If I hear about an illness I often think I have it myself	.605
10. If I have a bodily sensation or change I always wonder what it means	.347
11. I usually feel at moderate risk for developing a serious illness	.524
12. I often think I have a serious illness	.697
13. If I notice an unexplained bodily sensation I often find it difficult to think about other things	.588
14. My family/friends would say I worry too much about my health	.490
15. If I had a serious illness I would be almost completely unable to enjoy things in my life	.350
16. If I developed a serious illness … very small chance that ... medicine be able to cure me	.337
17. A serious illness would ruin almost every aspect of my life	.407
18. If I had a serious illness I would feel that I had lost quite a lot of my dignity	.372

Table B. Confirmatory Factor Analysis of the Italian Psychological Flexibility Dimension of the Multidimensional Psychological Flexibility Inventory (MPFI).

	Factor Loadings					
Items	Acceptance	Present Moment Awareness	Self as Context	Defusion	Values	Commited Action
1. I was receptive to observing unpleasant thoughts and feelings…	.566					
2. I tried to make peace with my negative thoughts and feelings…	.655					
3. I made room to fully experience negative thoughts and emotions…	.855					
4. When I had an upsetting thought…, I tried to give it space…	.859					
5. I opened myself to all of my feelings…	.731					
6. I was attentive and aware of my emotions		.806				
7. I was in tune with my thoughts and feelings…		.894				
8. I paid close attention to what I was thinking and feeling		.898				
9. I was in touch with … my thoughts and feelings		.892				
10. I strived to remain mindful and aware of my own thoughts…		.813				
11. Even when I felt hurt …, I tried to maintain broader perspective			.767			
12. I carried myself through tough … my life from a larger viewpoint			.860			
13. I tried to keep perspective even when life knocked me down			.890			
14. When I was scared or afraid, I still tried to see the larger picture			.904			
15. When something painful happened, I tried to take a balanced view…			.839			
16. I was able to let negative feelings come and go…				.881		
17. When I was upset, I was able to let those negative feelings pass through me…				.917		
18. When I was scared or afraid, I was able to gently experience those feelings…				.905		
19. I was able to step back and notice negative thoughts and feelings…				.548		
20. In tough situations, I was able to notice my thoughts and feelings…				.852		
21. I was very in-touch with what is important to me and my life					.793	
22. I stuck to my deeper priorities in life					.881	
23. I tried to connect with what is truly important to me on a daily basis					.883	
24. … I still tried to prioritize the things that were important to me					.862	
25. My deeper values consistently gave direction to my life					.802	
26. … I didn't quit working toward what is important						.837
27. Even when times got tough, I was still able to take steps toward what I value…						.897
28. …stressful, I still worked toward things that were important...						.919
29. I didn't let set-backs… in taking action toward what I really want in life						.868
30. I didn't let my own fears… get in the way of taking action toward my goals						.804

Table C. Confirmatory Factor Analysis of the Italian COVID-19 Peritraumatic Distress Index (CPDI).

Items	Factor Loading
1. Compared to usual, I feel more nervous and anxious	.752
2. I feel insecure and bought a lot of masks, medications, sanitizer, gloves …	.392
3. I can't stop myself from imagining myself or my family being infected and feel terrified …	.505
4. I feel empty and helpless no matter what I do	.778
6. I feel helpless and angry about people around me, governors, and media	.365
7. I am losing faith in the people around me	.473
12. I avoid watching COVID-19 news, since I am to scared to do so	.352
13. I am more irritable and have frequent conflicts with my family	.636
14. I feel tired and sometimes even exhausted	.703
15. Due to feelings of anxiety, my reactions are becoming sluggish	.804
16. I find it hard to concentrate	.725
17. I find it hard to make any decisions	.726
18. During this COVID-19 period, I often feel dizzy or have back pain and chest distress	.500
19. During this COVID-19 period, I often feel stomach pain, bloating, and other …	.526
20. I feel uncomfortable when communicating with others	.540
21. Recently, I rarely talk to my family	.398
22. I cannot sleep well. I always dream about myself or my family being infected by COVID-19	.369
23. I lost my appetite	.514
24. I have constipation or frequent urination	.387
